# Oncolytic virus immunotherapy: future prospects for oncology

**DOI:** 10.1186/s40425-018-0458-z

**Published:** 2018-12-04

**Authors:** Junaid Raja, Johannes M. Ludwig, Scott N. Gettinger, Kurt A. Schalper, Hyun S. Kim

**Affiliations:** 1 Division of Interventional Radiology, Department of Radiology and Biomedical Imaging, Yale School of Medicine, 330 Cedar Street, New Haven, CT 06510 USA; 2 Department of Diagnostic and Interventional Radiology and Neuroradiology, University Hospital Essen, University of Duisburg-Essen, Hufelandstr. 55, 45147 Essen, Germany; 3 Division of Medical Oncology, Department of Medicine, Yale School of Medicine, 330 Cedar Street, New Haven, CT 06510 USA; 4 Yale Cancer Center, Yale University School of Medicine, 330 Cedar Street, New Haven, CT 06510 USA; 5 Department of Pathology, Yale School of Medicine, 330 Cedar Street, New Haven, CT 06510 USA

**Keywords:** Oncolytic viruses, Oncolytic viral vaccine, Immunomodulatory oncolytic virus, Tumor niche biology, Cancer Immunoediting

## Abstract

**Background:**

Immunotherapy is at the forefront of modern oncologic care. Various novel therapies have targeted all three layers of tumor biology: tumor, niche, and immune system with a range of promising results. One emerging class in both primary and salvage therapy is oncolytic viruses. This therapy offers a multimodal approach to specifically and effectively target and destroy malignant cells, though a barrier oncoviral therapies have faced is a limited therapeutic response to currently delivery techniques.

**Main body:**

The ability to deliver therapy tailored to specific cellular targets at the precise locus in which it would have its greatest impact is a profound development in anti-cancer treatment. Although immune checkpoint inhibitors have an improved tolerability profile relative to cytotoxic chemotherapy and whole beam radiation, severe immune-related adverse events have emerged as a potential limitation. These include pneumonitis, pancreatitis, and colitis, which are relatively infrequent but can limit therapeutic options for some patients. Intratumor injection of oncolytic viruses, in contrast, has a markedly lower rate of serious adverse effects and perhaps greater specificity to target tumor cells. Early stage clinical trials using oncolytic viruses show induction of effector anti-tumor immune responses and suggest that such therapies could also morph and redefine both the local target cells’ niche as well as impart distant effects on remote cells with a similar molecular profile.

**Conclusion:**

It is imperative for the modern immuno-oncologist to understand the biological processes underlying the immune dysregulation in cancer as well as the effects, uses, and limitations of oncolytic viruses. It will be with this foundational understanding that the future of oncolytic viral therapies and their delivery can be refined to forge future horizons in the direct modulation of the tumor bed.

## Background

### Scope of Immuno-oncology

Medical oncology is in the midst of a massive paradigm shift: previously markedly toxic and poorly selective systemic chemotherapy and radiotherapy are now supplemented and in certain cases supplanted by more precise and sophisticated immunostimulatory therapies [1–3]. These strategies have shown improved overall survival in diverse tumor types and at different stages of progression, even in metastatic and previously incurable cancer [4]. The impact of this shift is proposed to be the most momentous to date in number of lives saved in person-years for advanced cancers. Notably, such treatments are able to induce up to total regression or remission [5, 6].

Intriguingly though the principle of immuno-oncology has long existed. Historically the first American immuno-oncotherapy dates to the late 1800s with the use of Coley’s toxin derived from bacterial exotoxins from *Streptococcus pyogenes* and *Serratia marcescans* that were injected into patients to treat solid tumors [7]. Since that time tremendous advances have been made. Current oncolytic viruses are now better tolerated, have comparable or superior effectiveness in achieving tumor response, and can be delivered through different approaches [8–10]. The ability to reintegrate anti-tumor immune surveillance, direct receptor stimulation or blockade to induce tumor apoptosis, or to specifically mark malignant cells as targets for destruction are three broad approaches of immunotherapy [2, 4, 6, 11–14]. Current anti-cancer immunotherapies consist of a wide range of strategies including the systemic use of monoclonal antibodies targeting co-regulatory pathways, small molecules, anti-tumor vaccines, cytokines, cell therapies and bacterial toxins (such as Coley’s toxin). Oncoviral therapies are emerging as a novel therapeutic class.

The superiority of oncoviral immunotherapy relative to other approaches relies in its specificity against tumor cells and not exclusively for targeting replicating cells. In addition, oncolytic viruses are less dependent on specific receptor expression patterns and the resultant mutational or transcriptional resistance that may occur. Oncolytic viruses can potentiate or restore already existing but ineffective anti-tumor immunity or induce a novel non-self antigen response.

### Mechanisms of Immunosurveillance

The mechanisms by which these immune therapies work on a cellular level include direct receptor-ligand signaling disruption, suppression of dominant tolerogenic pathways present in the tumor, and direct immune cell stimulation. The refinement of these immunomodulating and immune editing approaches to achieve full target specificity, induce lasting memory responses while maximizing tolerability has become the aspirational goal [1, 15]. The premise of using immunotherapy to treat malignancies is predicated on the cooperative function of less specific innate immune cells such as macrophages and natural killer (NK) cells; and specific primed lymphocytes tasked to surveil damaged and dysplastic cells and either mark them for phagocytosis, induce apoptosis or direct cytotoxic killing [5, 6, 16].

**Fig. 1:**
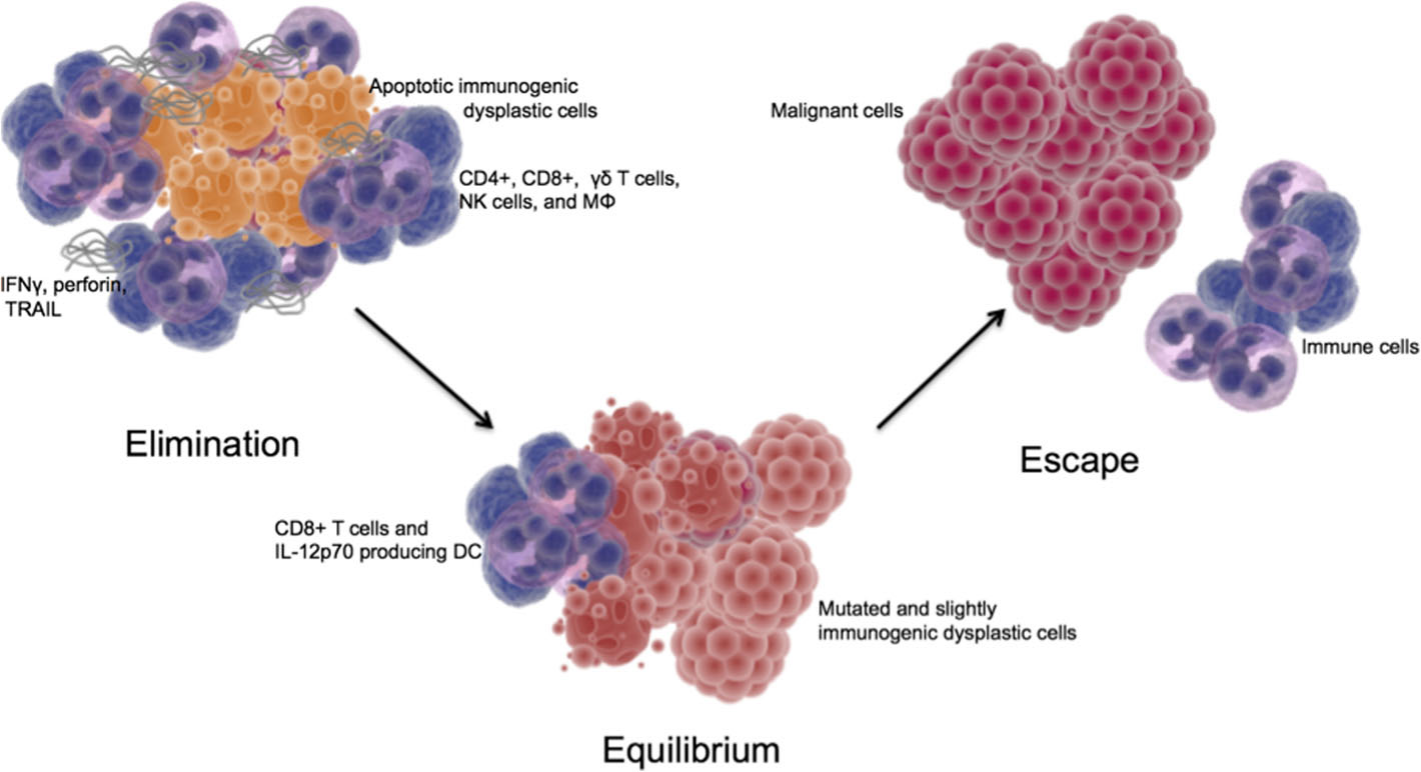
Cancer immunoediting with three phases. In the elimination phase the antitumor effector cells and macromolecules induce apoptosis and phagocytose the immunogenic dysplastic cells. In the equilibrium phase CD8+ T cells and dendritic cells maintain a homeostasis with further mutated and less immunogenic dysplastic cells. In the escape phase the immune cells do not recognize the malignant cells. Yellow: immunogenic dysplastic cells. Gray: antitumor macromolecules. Blue: immune cells. Red-orange: sculpted dysplastic cells. Red: malignant cells

This cancer immunoediting process includes three main stages: elimination, equilibrium, and escape. In the elimination phase there is early immune detection of malignant cells and clearance during which refinement or sculpting of the tumor by lymphocytes and glycoproteins can lead to the equilibrium phase, and then finally success in altered transcription for immune evasion or enter the escape phase [5, 17–19]. During the elimination phase there is continuous T-cell mediated eradication of malignant cells via effector responses including CD8+ T-cells, γδ T-cell subsets and NK cells, as well as macromolecules including IFNγ, perforin, and TNF-related apoptosis inducing ligands (Fig. 1) [11, 12, 16, 20–22].

In general, the traditional motif of antigen presentation to T and B cells eliciting both memory and effector cells are maintained in immunosurveillance of tumors. Multiple studies have demonstrated a survival benefit in tumors containing elevated numbers of lymphocytes and NK cells in a range of malignancies [23–26]. NK cells are able to recognize altered surface protein patterns and lyse tumor cells by co-stimulation with IL-2 regardless of prior sensitization [23, 27]. In the event that not all malignant cells are destroyed a functional homeostasis can result during which CD8+ T cells and IL-12p70 producing dendritic cells can limit the maximum number of tumor cells leading to a macroscopically dormant lesion [12, 28].

### Mechanisms of tumor escape

The development of any malignancy implies that transformed, atypical cells were able to escape scrutinization or killing by immune cells and disrupt this static state [28, 29]. Various mechanisms that have been postulated include upregulation of key tolerogenic pathways, mutation-based disruption of cellular proteins and receptors involved in tumor antigen presentation, dysregulation of effector responses and niche dysfunction. Increased expression of immune evasion targets include CD47, TGFβ, VEGF, IL-10, FLIP, FAS, and BCL X_L_, among others (Table 1) [19, 23, 28–36]. Altered expression of indoleamine 2,3 dioxygenase (IDO) in tumor cells or alternatively polarized/pro-tumorigenic macrophages can affect the local availability of tryptophan and kynurenine metabolites limiting T-cell function and also possibly modifying downstream effects of CTLA-4 signaling [28, 30]. Altered transcriptional downregulation or mutations associated with immune evasion include loss or reduction of potent proinflammatory mediators such as IFNγ, major histocompatibility complex/antigen presenting machinery and TNF related apoptosis inducing ligands and receptors.

**Table 1:** Common tumor escape associated changes

Molecule	Full Name	Level of Effect	Type of Disruption	Consequence	Ref
MHC	Major Histocompatibility Complex	Tumor	Downregulation	T cell anergy as costimulatory signal for epitope imprinting factor not presented to T cells	[31–33, 43]
TRAIL	Tumor Necrosis Factor Related Apoptosis Inducing Ligand	Tumor	Downregulation	Induction of NK cell apoptosis by TRAIL-R2 binding	[31, 32, 38, 39, 43]
FAS	CD95	Tumor	Downregulation	Inability to induce TNF superfamily mediated apoptosis	[31, 32, 37, 48]
HLA-E	Human Leukocyte Antigen E	Tumor	Upregulation	Binding to inhibitory receptor CD94/NKG2A on NK and CD8^+^ cells	[32, 44]
TGFβ	Transforming Growth Factor Beta	Tumor	Upregulation	Inhibition of CD8^+^ T cell and NK cell proliferation and differentiation and disruption of T cell stimulation by antigen presenting cells	[32, 34–36]
VEGF	Vascular Endothelial Growth Factor	Tumor	Upregulation	Inhibition of NK κ B mediated dendritic cell differentiation	[32, 43, 49]
IL 10	Interleukin 10	Tumor	Upregulation	Inhibition of dendritic cell differentiation and tumor cell TAP 1 and 2 production as well as CD4^+^ inhibition	[32, 42, 43]
FLIP	FLICE Inhibitory Pathway	Tumor	Upregulation	Inhibition of death receptor mediated apoptosis by caspase 8 and FADD binding	[32, 37]
CD47	Integrin Associated Protein	Tumor	Upregulation	Signal regulatory protein alpha stimulation within macrophages for ‘don’t eat me’ signal	[31]
Bcl X_L_	B Cell Lymphoma Extra Large	Tumor	Upregulation	Inhibition of TRAIL pathway and CD 95 mediated apoptosis	[32, 38]
IFNγ	Interferon Gamma	Tumor	Downregulation	Loss of STAT1 activation and resultant MHC production	[21, 31, 39, 41, 43]
IFNγ	Interferon Gamma	Niche	Upregulation	Induction of PDL1 production to induce T cell deactivation	[21, 31, 41, 43]
iMCs	Immature Myeloid Cells	Niche	Upregulation	Induction of T cell apoptosis, inhibition of T cell proliferation, induction of regulatory phenotype	[32]
Type II Mφ	Type II Macrophages	Niche	Upregulation	Disrupt Th1 immunity, promote angiogenesis and repair mechanisms	[21, 32, 45]
IDO	Indoleamine 2,3-dioxygenase	Immune	Upregulation	Suppression of activated cytotoxic T cells and induction of regulatory T cells	[30, 32, 50]
CD4^+^CD25^+^ T_reg_	Regulatory T Cells	Immune	Upregulation	Prevention of activation for CD4^+^, CD8^+^, and NK T cells	[35, 40]
CD1d restricted T cell	Type II Natural Killer T Cells	Immune	Upregulation	Suppression of cytotoxic T cell differentiation via TGFβ production	[32, 40]
PDL1	Programmed Death Ligand 1 or B7-H1	Immune	Upregulation	Induction of T cell apoptosis by binding of PD1	[32, 41]

Regarding niche effects, immune cell dysfunction such as T-cell anergy or inhibition can result as a consequence of the accumulation of CD4 + CD25+ Tregs and CD1d restricted T-lymphocytes [23, 30, 37–42]. Intriguingly, another proposed escape mechanism involves immature myeloid cells that, when clonally expanded, can suppress effector T-cell responses through multiple mechanisms including induction of apoptosis, inhibition of proliferation, or induction of a regulatory phenotype. Similarly anti-inflammatory macrophages (also referred to as “type 2” macrophages) at the tumor niche can act similar to immature myeloid cells to reduce antigen presentation and actively suppress adaptive anti-tumor responses [30, 43]. Robust clinical evidence about the critical role of immune surveillance in carcinogenesis and tumor progression stands from the observation that patients with primary or induced immunosuppression after organ transplants have a statistically significant increased risk of developing nearly every form of solid tumor [44, 45].

### Definition of an oncolytic virus

Conceptually similar to the seminal idea of Dr. Coley’s toxin, oncolytic viruses use attenuated viruses to infect tumor cells and generate de novo or boost pre-existing native immune response [7]. Most available oncolytic viruses are genetically modified to enhance tumor tropism and reduce virulence for non-neoplastic host cells [15]. Therefore, they can stimulate a proinflammatory environment by enhancing antigen release/recognition and subsequent immune activation to counteract the immune evasiveness of malignant cells. Indeed, oncolytic viruses also aim to harness or take advantage from the tumor’s tolerogenic mechanisms, which can facilitate viral infection and killing of cells that are not protected by the immune system [15]. This allows for a theoretical domino effect including chained viral transference between neoplastic cells and further immune activation.

There are presently numerous viral species in different stages of investigation for immuno-oncologic use. Possibly the best studied so far are Herpes viruses of which some strains have been found to have native tumor cell tropism while others have been engineered to improve selectivity [15, 46–48]. Initial explorations using herpes has shown promising results in murine glioblastoma [15]. Additional evidence has been seen in prostate cancer using a recombinant vaccinia and fowlpox virus able to upregulate prostate specific antigen and expression of three co-stimulatory factors involved in antigen presentation and T-cell activation [12, 13, 49, 50]. Moreover, various strains of recombinant vaccinia virus have shown promise as antineoplastic agents. One strain has demonstrated tumor anti-angiogenesis, another has shown efficacy against hepatocellular carcinoma in animal models and the third improves tumor cell recognition [51–54]. Other viruses that have been or are being explored as possible vehicles for immunomodulation in cancer include Newcastle Disease Virus, coxsackie, reovirus, and even measles (Table 2) [15, 48, 55–57].

**Table 2:** Viruses currently under consideration for oncoviral therapy

Herpes Simplex Virus	Mumps	Retrovirus
New Castle Disease Virus	Moloney Leukemia Virus	Parvovirus
Reovirus	Adenovirus	Seneca Valley Virus
Measles	Vesicular Stomatitis Virus	Vaccinia
Fowlpox	Coxsackie Virus	

### Scope of oncolytic viruses

At present the only FDA approved oncolytic viral therapy is talimogene laherparepvec (T-Vec or Imlygic) for use in metastatic melanoma, though there are numerous other viruses being developed pre-clinically and clinically. As of 2016 there are reportedly at least eight oncolytic viruses in phase I, nine in phase II, and two in phase III clinical trials [58, 59]. Notably, the therapeutic potential of oncolytic viruses is way beyond melanomas and current studies are ongoing at least in pancreatic and hepatocellular carcinomas. In fact, a search of all registered clinical trials in 2017 demonstrates 78 interventional trials referencing the usage of an “oncolytic virus” and spanning nearly every solid organ malignancy (Table 3) [60]. This ability for near universal therapeutic impact in cancer makes oncolytic viruses a unique therapeutic tool. While more traditional therapies such as chemotherapy and radiotherapy lack tumor specificity targeting all replicating cells, and other immunotherapies have limited scope by relying on the presence of a specific ligand/receptor, oncolytic viruses are postulated to be both specific to neoplastic cells and have an expansive immunostimulatory latitude. The broad impact of oncolytic viruses is the consequence of using of the host adaptive immune response that is able to sharply distinguish target and non-target cells for precise specificity; while also being able to harness signals ubiquitous to perhaps all malignancies.

**Table 3:** Current and recently completed trials using oncolytic viruses

Virus	Strain	Manufacturer	Phase	Targeted Malignancy	Primary or Adjuvant Therapy
Herpes Simplex Virus I	Talimogene Laherparepvec (T-Vec)	Amgen	I/II	Breast	Adjuvant
			II	Melanoma	Primary
			I	Pancreatic	Primary
	TBI-1401(HF10)	Takara	I	Superficial Solid Tumors	Primary
			II	Melanoma	Adjuvant
	G207	MediGene	Ib/II	Glioma	Primary
	HSV1716	Virtu Biologics	I/II	Mesothelioma	Primary
			I	Bone, Sarcomas, Neuroblastomas	Primary
Adenovirus/Herpes Simplex Virus	ADV/HSV-tk	Merck	II	Breast and NSCLC	Adjuvant
Adenovirus	LOAd703	Lokon	I/II	Pancreatic	Adjuvant
	CG0070	Cold Genesys	II	Bladder	Primary
	ColoAd1(Enadenotucirev)	PsiOxus	I	Colorectal, NSCLC, Bladder, and Renal Cell	Primary
			I/II	Colorectal, Bladder, and Epithelial	Primary
			I	Ovarian	Primary
	ONCOS-102	Targovax Oy	I	Advanced Solid Tumors	Adjuvant
			I	Melanoma	Adjuvant
	DNX-2401	DNAtrix	II	Brain	Adjuvant
	VCN-01	VCN	I	Advanced Solid Tumors	Adjuvant
			I	Pancreatic	Adjuvant
	Ad-MAGEA3 and MG1-MAGEA3	Turnstone	I/II	NSCLC	Adjuvant
			I/II	Advanced Solid Tumors	Primary
	NSC-CRAd-Survivin-pk7	Northwestern	I	Glioma	Adjuvant
	Ad5-yCD/mutTKSR39rep-hIL12	Henry Ford	I	Prostate	Adjuvant
	Ad5-yCD/mutTKSR39rep-ADP	Henry Ford	I	NSCLC	Primary
Measles	MV-NIS	Mayo	I	Breast and Head and Neck	Primary
			I/II	Ovarian	Primary
			I	Nerve Sheath	Primary
			I	Mesothelioma	Primary
			I/II	Multiple Myeloma	Adjuvant
	MV-NIS	University of Arkansas	II	Multiple Myeloma	Adjuvant
Vaccinia	GL-ONC1	Genelux	I	Advanced Solid Tumors	Primary
			I	Head and Neck	Primary
			Ib	Advanced Solid Tumors	Adjuvant
			I	Ovarian	Primary
	Pexastimogene Devacirepvec (Pexa-Vec)	Jennerex	III	Hepatocellular	Adjuvant
			I/IIa	Colorectal	Adjuvant
			I	Advanced Solid Tumors	Adjuvant
			I	Blue Cell	Primary
			I	Melanoma, Lung, Renal Cell, Head and Neck	Primary
Reovirus	REOLYSIN	Oncolytics	I	Colorectal	Adjuvant
			Ib	Bladder	Adjuvant
			Ib	Pancreatic	Adjuvant
			I	Multiple Myeloma	Adjuvant
			Ib	Plasma Cell Cytoma	Adjuvant
			II	Ovarian and Peritoneal	Adjuvant
Coxsackievirus	CVA21(CAVATAK)	Viralytics	II	Melanoma	Primary
			I	NSCLC	Adjuvant
Parvovirus	H-1PV(ParvOryx)	Oryx GmbH	I/IIa	Glioblastoma Multiforme	Primary
Polio/Rhinovirus	PVSRIPO	Duke	I	Glioma	Primary
Vesicular Stomatitis Virus	VSV-hIFNbeta-NIS	Mayo	I	Endometrial	Primary

T-Vec is a genetically manipulated Herpes Simplex Virus 1 (HSV-1) with an affixed granulocyte macrophage colony-stimulating factor (GM-CSF) [15, 61–63]. The virus is locally delivered but can produce recruitment of T-cells in distant non-injected metastases [15, 47, 64–66]. T-Vec has exhibited remarkable success with up to a 15% complete regression of injected lesions in patients with metastatic melanoma, the primary population in which the virus has presently been attempted [15, 47, 61–66].

### Immunomodulatory mechanisms of oncoviral therapy

**Fig. 2:**
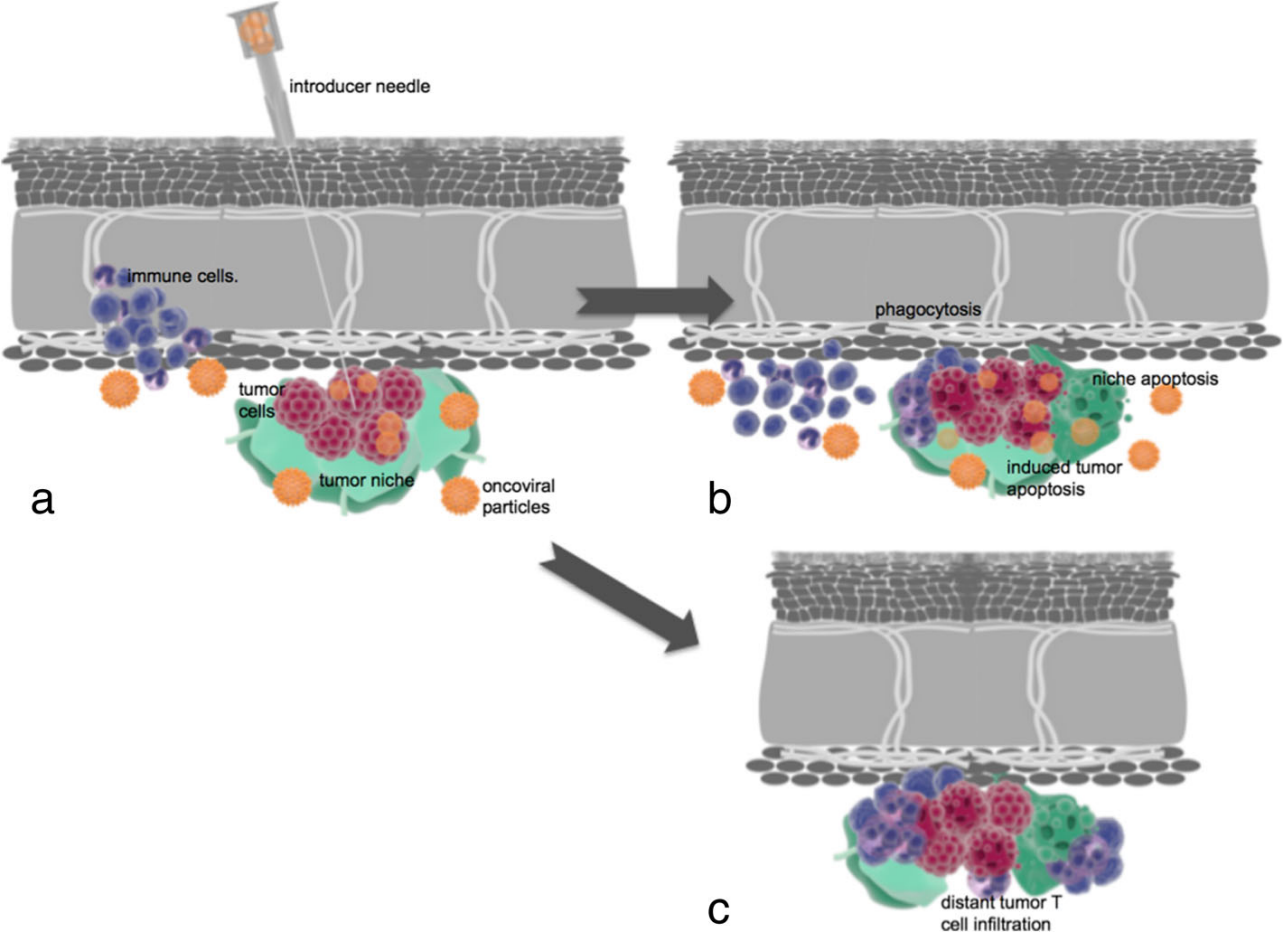
**a** Intratumoral inoculation of an oncolytic virus with transfection and early immune cell recruitment. **b** Advanced transfection of an oncolytic virus into tumor and niche cells with induction of immune cells resulting in apoptosis, direct cell lysis, niche disruption, and phagocytosis**. c** Distant tumor immune infiltration induced by local immune conditioning. Blue: immune cells. Red: tumor cells. Orange: oncoviral particles. Green: tumor niche

Similar to other immunotherapies oncolytic viruses have a multimodal mechanism of action with both direct and indirect toxic effects on tumor cells such as autolysis, immune cell honing, destruction of vascular supply and potentiation of other adjunctive anti-cancer therapies (Fig. 2) [15, 48].

Direct cell lysis from traditional anti-viral machinery is one method of toxic injury and is postulated to be dose dependent with excellent tolerability even at high doses [15, 57]. For instance, infected cells can instigate an interferon or Toll-like receptor response by transcribing antigens that are then transited to the cell surface or detected by intracellular components of Toll-like receptors. These antigens, termed pathogen associated molecular patterns (PAMPs), can be the viral capsid, nucleic acids, or proteins. Immune recognition of virally infected cells can initiate a cascade using TNF and IFN related factors as well as retinoic acid inducible gene 1 to stimulate the JAK/STAT pathway that gives positive feedback to IFN to activate protein kinase R. The latter senses intracellular viral material and stops protein transcription ultimately promoting apoptosis and viral clearance [67]. Additionally, infected cells display transcription of cytokines and other proinflammatory signaling peptides [15, 68]. For instance HMGB1, calreticulin, and viral/cellular DNA can be released in the tumor microenvironment and elicit immune-cell recruitment [47, 69, 70]. Some of these anti-viral signaling mechanisms involve selective upregulation of peptide and siRNAs. These responses are not observed in non-tumor host tissue cells [71]. Another mechanism, as is seen in coxackievirus targeting non-small cell lung cancer, comprise specific viral antigen proliferation disrupting essential cell survival pathways (in this case B3 Ag disrupting ERK/MEK) [55]. Cytometric analyses have also shown upregulation of immunotherapeutic targets such as CTLA-4 in tumor infiltrating T-cells, suggesting a possible role of oncolytic viruses in neo-adjuvant/adjuvant treatment along with systemic immunotherapies [64].

### Vaccine mechanism of oncoviral therapy

**Fig. 3:**
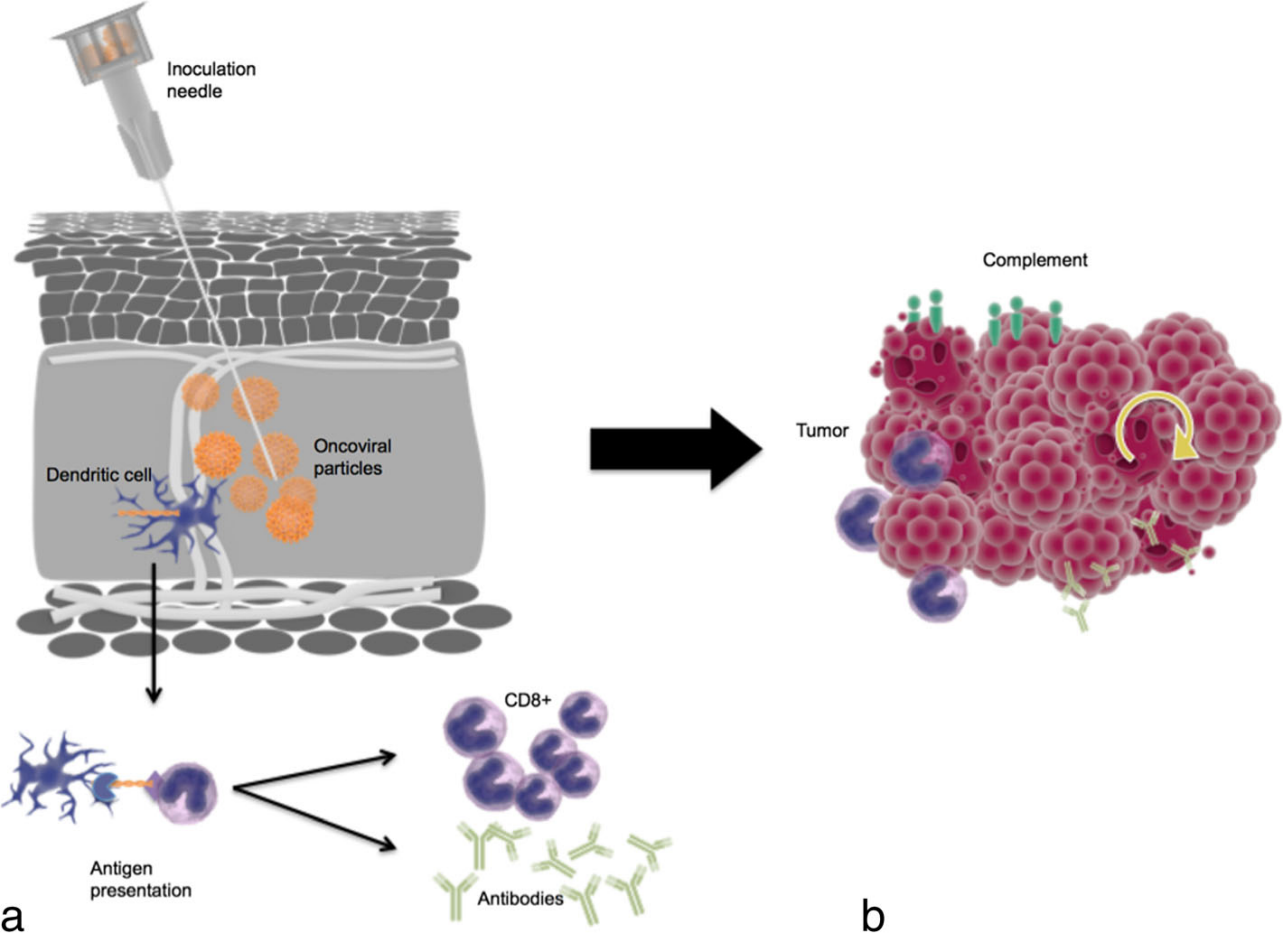
**a** Inoculation of the oncoviral vaccine with antigen detection by dendritic cells and presentation to CD4+ and CD8+ lymphocytes with clonal expansion and antibody formation. **b** Induction of immune storm by cytotoxic T cell invasion, antibody mediated destruction, and complement formation with feedback autophagy and apoptosis. Orange: oncoviral vaccine. Blue: immune cells. Light green: antibodies. Teal: Complement

The concept of tumor vaccination has existed for some time; however, the mechanistic considerations of how to effectively prime and activate the immune system against tumor cells have not translated into major clinical success. The underlying physiology of this process consists of immune conditioning and generation of memory T-cell responses by exposing antigens that are expressed robustly and specifically in the target tissue. The use of viruses to deliver antigens is beneficial as the encoded genetic material is well conserved during infection and subsequent translation. In particular, a multifaceted response to tumor antigens released following necrosis and apoptosis results from exposure to PAMPs, danger associated molecular patterns (DAMPs: such as heat shock proteins, uric acid, calreticulin, HMGB-1), and cytokines (such as IFN 1, interleukin 12, and TNF α). Consequent to this, vigorous antigen presenting cell maturation occurs which then cascades to CD4+ and CD8+ T-cell activation. CD4+ and CD8+ T-cell responses can mediate global anti-tumor effects at distant loci and direct tumor cell killing [67]. Immune conditioning has been explored as in the case of Newcastle Disease Virus transfection in IFN-depleted lung tumor cells which can modulate genetic transcription of IFN *β* [56]. Additional studies in animal models and early human trials have shown that oncolytic viruses can produce antibody mediated, complement dependent, and tumor-cell specific cytotoxicity. The consequences of this include triggering of autophagy or apoptosis, recruitment of lymphocytes and phagocytic cells, and direct toxic injury from inflammatory cytokines [15, 68]. This has previously been described as creating an “immune storm” within a tumor to augment antigen recognition that can lead to lesion debulking and facilitate adjuvant therapies (Fig. 3) [14, 15, 61, 72]. Moreover, this can theoretically be further harnessed and tailored to target tumors by genetic manipulation [15, 68]. Consequently the use of an oncolytic virus can be used as an effective tumor vaccine.

There are host factors predictive of oncoviral therapeutic success. The strongest favorable predictor of immunotherapeutic response in human and animal models is the pre-existence of tumor infiltrating lymphocytes as well as high tumor expression of immunomodulating targets prior to inoculation. Among these, upregulation of type I IFN has been recognized as the top marker associated with sensitivity to immunostimulatory agents [64, 73, 74]. In addition, emerging research suggests that the dissimilar immune cell composition across different tissues may influence tumorigenesis and therapeutic response [75, 76]. Variation in constituent microenvironmental niche features including intercellular signaling, extracellular components, and nutrients may be directly involved. To date, nearly all organs have been described to contain unique “tissue-resident memory T cells (T_RM_)” which are either of a CD4 or CD8 lineage. These immune cells are, as their name implies, restricted in location to a single, frequently non-lymphoid, organ and are believed to arise from the primary response to antigens [75]. These cells serve as a type of local sentry that is biochemically familiar to its surrounding tissues and can rapidly stimulate an immune reaction when a non-resident antigen is detected. Of course, as with other immune cells, the ability of T_RM_ to recognize a tumor is dampened during immune evasion. However, the precursor T_RM_ cells have the potential to be primed against a tumor when provided the appropriate stimulus such as from a tumor derived dendritic cell [75]. This concept has been demonstrated in the skin and genitourinary tract where local administration of a vaccine has led to the induction of T_RM_ cells against tumors to enhance therapeutic response [75].

Additionally, different tissues also have variable antigenic exposure patterns. The most prominent example of this is the liver which, as a central organ of metabolism, has a large filtration component as well as a dual blood supply. Antigenic exposure in the liver includes > 100 times higher concentrations of microbe associated molecular patterns in comparison to peripheral blood and high concentrations of DAMPs. These are then widely exposed to the body’s largest population of tissue resident macrophages (Kupffer cells) as well as NK cells, and transiting and resident T lymphocytes [76]. Consequently, the sensitivity of the liver to immune stimulation would likely contrast in gradient to the lung, colon, adrenal glands, muscle, and other organs with distinct antigen exposure. It has been posited that this local antigenic landscape is partially a limiting factor in hereto-limited success of systemically administered vaccination with tumor antigens and that the major histocompatibility and T-cell receptor complex may require co-activation with local chemokines or resident immune cells. At least in theory, oncolytic viruses can affect the antigenic profile of the injected tissue by inducing not only an anti-tumor immune response but also an anti-viral reaction against the antigenic viral components [76]. The significance of each of these considerations from a clinical perspective remains to be investigated as are any potential solutions.

### Oncolytic viruses as adjuvant therapy

Another avenue by which oncolytic viruses can impact oncologic care is by functioning as a therapeutic adjuvant. Concomitant administration with other therapies may have two primary mechanisms: augmenting other immunotherapeutics and overcoming primary resistance patterns.

The enhancement of other immunotherapies is potentially mediated by the creation of a pro-inflammatory milieu able to upregulate the targets for additional interventions such as co-regulatory checkpoint blockade. Consistent with this notion, CTLA-4 and PD-L1 are known to be increased at and mediate peripheral immune tolerance upon inflammation or tissue damage. Adjuvant administration of oncolytic viruses upregulate the expression of pro-inflammatory cytokines such as IFN*γ* which would in turn increase JAK 1/2 signaling and antigen expression to augment tumor response to checkpoint blockade [77–79]. This has been shown to be clinically beneficial in initial trials where an adjunctive oncolytic virus with CTLA-4 or PD-1 inhibition was superior to either monotherapy [80, 81]. Furthermore, early phase clinical trial suggest oncolytic viruses in conjunction with PD-1 inhibition can mold the tumor cell niche to be more susceptible to other non-immune anti-cancer treatments [82]. Patients showing tumor response when treated with these agents display typically higher tumor-infiltrating lymphocyte counts (independent of baseline level) as well as upregulation of PD-L1 and IFNγ [83].

Additionally the issue of primary and acquired immunotherapeutic resistance has become a prevalent concern that may be addressed by oncolytic viruses. Using the example of PD-1 axis inhibition, some estimates note that up to one out of four (25%) patients with melanoma who initially responded to PD-1 axis blockade develop resistance that is clinically evident as disease progression within two years of treatment [77, 84]. Hypothesized mechanisms of resistance include genetic loss of *β*2 microglobulin, reduced tumor infiltrating lymphocytes, antigen loss, signaling disruption, ineffective CD8+ T-cell function, upregulation of alternative immune checkpoints, or loss of downstream signaling via JAK1/2 gene modifications [85–87]. However, the IFN I pathway does appear to remain intact for many of these patients [77–79]. This has been postulated as a possible oncoviral bypass to restitute sensitivity in patients who develop resistance [64, 77].

### Systemic effects of oncoviral therapy

An intriguing finding in the study of oncolytic viruses has been the effects on distant metastases in patients with locally inoculated lesions, a phenomenon commonly known as “abscopal” effect. The range of oncolytic viral transfection is unquestionably limited to a loco-regional distribution as has been demonstrated in multiple animal and human models where metastatic lesions have been sampled and proven to be absent of viral DNA or RNA. However, the impact of oncolytic viruses has been found to extend to loci devoid of virus causing regression or delayed tumor growth [15, 64–66, 88, 89]. It is unclear how this effect occurs and whether it is mediated directly by an unidentified and yet unmeasured viral product, by crossed-antigenic reaction or as a consequence of global immune conditioning/stimulation. Although recruitment of tumor infiltrating lymphocytes to distant uninjected metastatic sites after oncoviral injection has been consistently documented [15, 64–66, 88, 89], the characteristics of the immune response differ from that of the primary site. One animal study illustrated the infiltration of CD8+ and CD 4+ T-cells at the remote lesions in an IFN I dependent manner though regulatory T-cells were absent despite being noted at the site of inoculation [64].

### Current approaches to delivery of oncolytic viruses

One of the greatest challenges for effective oncoviral therapy has been sufficient drug delivery. There is exceptionally poor bioavailability of systemically administered oncolytic viruses. Moreover, even in the case of intravenous delivery the host immune system rapidly sequesters and degrades the attenuated virus through the reticuloendothelial system lead by red pulp macrophages in the spleen and Kupffer cells of the liver [15, 68, 90]. Viral particles are opsonized by antibodies, complement, and other factors to enhance endothelial cell and macrophage binding and phagocytosis [15, 91]. Of note, there are no reports of poor dose tolerance to oncoviral therapy or reverted virulence by the inactivated particulates. Balancing the degree of local immunosuppression provides a complex challenge in oncoviral therapy. On one end immunosuppression can increase intratumoral distribution of the therapy. Conversely, augmentation of the host immune system will enhance targeting of transfected tumor cells but the intratumoral viral spread will be pruned [15]. Consequently and to date, the only route by which oncoviral therapies have been delivered in sufficient quantity to be clinically efficacious is via loco-regional or direct inoculation [15, 47, 68, 90].

### The role of image guidance in oncoviral therapy

Future success and broad use of oncoviral therapy is naturally bound to image guided delivery. As has been elsewhere described the concept of image guidance is expansive and includes planning, targeting, controlling, monitoring, and assessing treatment response for lesions and each of these tasks is integrally important to the success of the therapy [92]. Image review for planning is an essential step not only to locate the neoplastic lesions but also to characterize and prioritize targets for therapeutic delivery. For instance the identification of a lesion that is large but necrotic would not be preferred over one that is smaller but demonstrates features of active metabolism/proliferation. The rationale for this is that functional cells are required for viral transfection and immune cell recruitment and these tissues can also be sampled to assess tumor response. Proposed needle trajectory can also be mapped via imaging to minimize crossing unwanted or high-risk anatomical structures. Furthermore, image guidance enables direct access to remote body locations that would not necessarily be amenable to effective hematogenous distribution of systemic therapy such as malignancies with low mitotic indices or that are poorly vascularized.

However, even in well vascularized tumors blood vessels have been described as imperfectly synthesized with pitfalls including unusual or absent branching patterns, irregular shape and contour, and hyperpermeability each of which can further limit systemic drug delivery [93–96]. Also as outlined above oncoviral therapy via alternative routes is typically sequestered, denatured, and cleared by the host immune response or lymphatics particularly in the liver and spleen [15, 68]. Nonetheless, image-guided delivery is able to circumvent this barrier and maximize the local virus availability and potential efficacy by direct visualization the locus in which it was administered. An additional benefit of image guided needle system based delivery of oncolytic viruses includes possibilities for monitoring of the target lesion with morphologic and molecular analyses. That is, image guidance is used to place a large bore needle into the target site through which biopsy can be performed at the time of therapy. These samples can then be analyzed for constituent composition of tumor cells and profile, immune cells (e.g. resident memory T cells), and the local microenvironment (e.g. gene expression microarrays).

Imaging approaches for therapeutic delivery may include any form of cross-sectional imaging though, for similar considerations as with other locoregional therapies, ultrasound and computerized tomography are likely to be the most favored. Ultrasound can allow for real-time, dynamic, non-ionizing radiation based imaging of the target lesion, the introducer and biopsy needles and architectural distortion from obtaining a sample and instilling the therapy. However, ultrasound is limited by patient factors such has habitus and by location of a target lesion as well as imaging characteristics as lesions can be isoechoic to and hence “invisible” in their surroundings by ultrasound. CT in comparison is favorable for deeper lesions as well as lesions isoechoic to their surroundings and those that may benefit from contrast enhancement. MRI may also be considered as a potential imaging mechanism though the procedure time, cost, and need to exclude metallic tools would be prohibitive.

Specific technical approaches may vary based on patient factors and tumor anatomy though the general technique would likely entail image guided placement of a large bore guide needle into a non-necrotic portion of the tumor. Once satisfactorily positioned, a biopsy and hand-injection may be performed and, if necessary, the guide needle can be repositioned to treat additional regions of the tumor.

### Advantages of direct inoculation

Furthermore, the inoculation of the virus directly into lesion would enable favorable pharmacokinetics. These benefits include maximization of the drug concentration at the target lesion with a lower dose where they would be maximally retained and would limit elimination. Selection of which index and non-index lesions to inoculate is another benefit as is more precise dose adjustments into individual lesions as possible with direct inoculation as the delivery would be to the targeted site alone. Similarly, optimization of timing of delivery as neoadjuvant, adjuvant, or primary therapy could also be achieved. The clinical benefit of intratumoral injection delivery for oncoviruses has already been demonstrated for local and potential systemic anti-tumor response in the T-VEC OPTIM Phase III clinical trials [67].

Direct injection enables the prospect of delivering therapy via novel or unique vehicles such as polymeric micelles, nanoparticles or as implants. Image guided therapy would be by far the most resource-efficient modality as there would be negligible waste or loss of therapy given the image directed planning and localization of the target lesion. With respect to monitoring there is a role for both direct and indirect approaches. Direct imaging of intratumoral distribution of viral products has been achieved in herpetic viruses via HSV thymidine kinase phosphorylation and intracellular sequestration of positron emitting substrates [15, 97]. Gene splicing with thyroidal sodium iodide symporter has also been performed in animal models with iodinated and technetium based media to monitor distribution of oncoviral transcription within hosts, a concept validated with an adenovirus via pertechnatate based SPECT imaging [15, 68, 98].

### Potential limitations

As with all procedures, there is of course an associated risk with image guided oncoviral therapy. However, the overall risks are quite low and comparable to related standard of care procedures. Risks may be categorized as those related to technique and therapy. From a technical standpoint bleeding and inadvertent organ injury are the major potential adverse events and are deemed extremely unlikely. These risks are identical to the risk accepted in biopsying a mass that is at times standard of care for the targeted lesion. Unlike other locoregional therapy considerations such as thermal injury and electric neural conduction, direct oncoviral therapy does not require additional precautions. Moreover, regarding the risk of the therapy itself, as previously mentioned there are no reported cases of reverted virulence of the virus. Local inflammatory reaction is of course possible and to an extent desired with theoretical risk of a deregulated inflammatory response, though again, no current reports of this exist.

One additional risk specific to oncolytic viral therapy would be material leakage through the needle tract, though the likelihood of this is low as the inner diameter of an 18-gauge needle is less than one millimeter. Still this is a valid consideration and even though oncolytic viruses do not have systemic effects a local reaction could in principle occur. Approaches to minimize this if the risk achieves clinical significance could include track patching with autologous blood as is used for some lung and liver biopsies or using a needle system that performs tract ablation.

Additionally, even with direct inoculation there is a potential for neutralizing antibodies and tumor niches can be immune suppressive both of which can dampen therapeutic responses [76]. Furthermore, efficacy of oncoviruses may be limited by the tumor niche if the tumor cells are suspended in growth phase in response to hypoxia or acidosis or from nearby necrosis, calcification, or high interstitial pressure. Also an oncovirus that too rapidly induces apoptosis can also be disadvantageous as an optimal quantity of daughter viruses may not have been replicated [67, 76]. Acquired resistance or tumor adaptation to oncolytic viruses or associated tumor immune pressure is also a possibility.

### Distal effects of locally inoculated oncolytic virus

Image guided inoculation offers the prospect of superior tolerability as the viral product would be localized. As described previously, studies have demonstrated the paucity of viral products available at remote loci. However, there are systemic immune responses documented away from the injection site [15, 64–66, 88, 89]. This does increase the prospect of adverse effects, though this too is tempered compared to systemic therapy, as the theoretical reaction would be immune mediated and cross priming of immune activation would be specific to the area of insult (i.e. the inoculated tumor). Finally, the assessment of response to therapy can of course be performed via diagnostic radiographic means but also by biopsy assessments of tumors to analyze cellular level changes and response to therapy. This will provide extremely valuable feedback to interventionalists, as it will guide future decision-making regarding therapeutic planning for future patients.

### Future prospects

In the time of new and promising immuno-oncologic therapies, image-guided oncoviral therapy offers another avenue of hope for patients with previously unresectable, advanced malignancies not amenable to other classical oncologic therapies. The idea of image directed, locally delivered, molecular therapy supplemented by immune conditioning by which the delivered particles induce an indirect native tissue response is a patient-centered and personalized approach. Here in the context of oncolytic viruses we discussed recruitment of immune cells and also modification of adjacent niche cells. This concept though may be extended to other host cell processes. That is to say that modulation of a tissue’s microenvironment via image-targeted biotherapeutics may allow in the future not only oncotherapy but also controlled disruption of localized autoimmune phenomena, dampening of transplant induced immune reactions and even facilitate conditions for reparative or regenerative tissue construction.

## Conclusion

The evolution of oncologic therapies has led to increasingly targeted and nuanced regimens that seek to impose maximal impact on malignant cells, while simultaneously sparing collateral non-tumor tissues and minimizing adverse effects. This is most prominent in the rapid development within the realm of immunotherapy where the preponderance of efforts to date has utilized systemic agents. However, as presented above, oncoviral therapies represent another option for immune stimulation acting locally  to drive potent anti-tumor immune effects. This form of immunomodulation may herald another phase in anti-cancer immunotherapy with less toxicity, increased specificity, and hopefully improved survival.

## Abbreviations

BCL X_L_: (B cell lymphoma extra large)
CD1d: (Cluster of differentiation 1d)
CD25: (Cluster of differentiation 25)
CD4: (Cluster of differentiation 4)
CD47: (Cluster of differentiation 47)
CD8: (Cluster of differentiation 8)
CTLA 4: (Cytotoxic T lymphocyte associated protein 4)
DNA: (Deoxyribose nucleic acid)
ERK/MEK: (Extracellular signal regulated kinase/mitogen activated protein kinase extracellular signal related kinase kinase)
FAS: (CD 95)
FLIP: (FLICE inhibitory pathway)
GM-CSF: (Granulocyte macrophage colony stimulating factor)
HMGB-1: (High motility group box protein 1)
HSV-1: (Herpes simplex virus 1)
IDO: (Indoleamine 2,3 dioxygenase)
IFN γ: (Interferon gamma)
IL 10: (Interleukin 10)
IL 2: (Interleukin 2)
JAK 1/2: (Janus associated kinase 1/2)
PD-1: (Programmed death 1)
PD-L1: (Programmed death ligand 1)
siRNA: (Short ribose nucleic acid)
SPECT: (Single photon emission computed tomography)
TNF: (Tumor necrosis factor)
T_RM_: (Resident memory T cells)
T-Vec: (Talimogene laherparepvec)
VEGF: (Vascular endothelial growth factor)
